# Engineered living photosynthetic biocomposites for intensified biological carbon capture

**DOI:** 10.1038/s41598-022-21686-3

**Published:** 2022-11-04

**Authors:** Pichaya In-na, Elliot B. Sharp, Gary S. Caldwell, Matthew G. Unthank, Justin J. Perry, Jonathan G. M. Lee

**Affiliations:** 1 School of Engineering, Newcastle University, Merz Court, Claremont Road, Newcastle upon Tyne, NE1 7RU UK; 2 Department of Applied Sciences, Faculty of Health and Life Sciences, Northumbria University, Newcastle upon Tyne, NE1 8ST UK; 3 School of Natural and Environmental Sciences, Newcastle University, Ridley Building, Claremont Road, Newcastle upon Tyne, NE1 7RU UK; 4 Present Address: Department of Chemical Technology, Faculty of Science, Chulalongkorn University, Bangkok, Thailand

**Keywords:** Environmental sciences, Chemistry, Polymer chemistry, Microbiology, Bacteria, Biofilms, Industrial microbiology, Climate sciences, Atmospheric science

## Abstract

Carbon capture and storage is required to meet Paris Agreement targets. Photosynthesis is nature’s carbon capture technology. Drawing inspiration from lichen, we engineered 3D photosynthetic cyanobacterial biocomposites (i.e., lichen mimics) using acrylic latex polymers applied to loofah sponge. Biocomposites had CO_2_ uptake rates of 1.57 ± 0.08 g CO_2_ g^−1^_biomass_ d^−1^. Uptake rates were based on the dry biomass at the start of the trial and incorporate the CO_2_ used to grow new biomass as well as that contained in storage compounds such as carbohydrates. These uptake rates represent 14–20-fold improvements over suspension controls, potentially scaling to capture 570 tCO_2_ t^−1^_biomass_ yr^−1^, with an equivalent land consumption of 5.5–8.17 × 10^6^ ha, delivering annualized CO_2_ removal of 8–12 GtCO_2_, compared with 0.4–1.2 × 10^9^ ha for forestry-based bioenergy with carbon capture and storage. The biocomposites remained functional for 12 weeks without additional nutrient or water supplementation, whereupon experiments were terminated. Engineered and optimized cyanobacteria biocomposites have potential for sustainable scalable deployment as part of humanity’s multifaceted technological stand against climate change, offering enhanced CO_2_ removal with low water, nutrient, and land use penalties.

## Introduction

Climate change poses an existential threat to global biodiversity, ecosystem stability and to humanity. To curtail its worst effects will require coordinated and extensive decarbonization programs and will certainly need some form of direct removal of greenhouse gases from the atmosphere^1^. Despite positive indicators towards decarbonizing electricity generation^2,3^, there are yet no economically sustainable technological solutions for the drawdown of atmospheric carbon dioxide (CO_2_)^4^; although capture from flue gas is progressing^5^. In lieu of scalable and practical engineered solutions, humanity must look to nature’s own carbon capture engineers; photosynthetic organisms (phototrophs). Photosynthesis is nature’s carbon capture technology; however, its capacity to reverse anthropogenic carbon enrichment within a meaningful timescale is equivocal, with enzymatic inefficiencies and the capacity for deployment at an appropriate scale being questioned. One potential vehicle for phototrophy is through afforestation, with the trees harvested for use in bioenergy with carbon capture and storage (BECCS) which, as a negative emissions technology, can contribute to reducing net CO_2_ emissions^1^. However, to reach the 1.5 °C Paris Agreement target, BECCS as the main approach would consume between 0.4 and 1.2 × 10^9^ ha of land, equivalent to 25–75% of current global cropland^6^. Further, uncertainties surrounding the global CO_2_ fertilization effect cast doubt on afforestation’s potential overall effectiveness^7^. If we are to meet the temperature targets set by the Paris agreement, greenhouse gas removal (GGR) from the atmosphere is required on the scale of 100 s of Gt_CO2_ per year. Recently, UK Research and Innovation announced funding for five GGR projects^8^ including management of peatlands, enhanced rock weathering, tree planting, biochar, and the growth of perennial crops for feeding to a BECCS process. To remove in excess of 130 Mt_CO2_ per annum from the atmosphere would cost 10–100 US$/t_CO2_ at 0.2 to 8.1 Mt_CO2_ per annum for peatland restoration, 52–480 US$/t_CO2_ at 12–27 Mt_CO2_ per annum for rock weathering, 0.4–30 US$/t_CO2_ at 3.6 MtCO_2_ per annum with a 1% increase in woodland area, 0.4–30 US$/t_CO2_ at 6–41 Mt_CO2_ per annum for biochar, and 140–270 US$ per t_CO2_ at 20–70 Mt CO_2_ per annum for perennial crops with BECCS^9^.

Taken together these methods have the potential to meet the target of 130 Mt_CO2_ per annum but the cost of rock weathering and BECCS are high and, biochar whilst being relatively inexpensive and having no land use issues, requires a feedstock for the process that generates the biochar. This suggests that there is scope to develop and deploy other GGR technologies.

Rather than looking to the land for solutions there is just cause to look to the water; specifically, to unicellular phototrophs such as microalgae and cyanobacteria^10^. Algae (including cyanobacteria) fix approximately 50% of global CO_2_, despite contributing only 1% of global biomass^11^. Cyanobacteria are nature’s original bio-geoengineers that, through oxygenic photosynthesis, laid the very foundations for respiratory metabolism and the evolution of multicellular life^12^. The idea of applying cyanobacteria for carbon capture is not new^13^; however, innovative approaches towards their physical deployment are opening new horizons for these most ancient organisms.

Open ponds and photobioreactors are the default assets when using microalgae and cyanobacteria for industrial applications. These culture systems exploit suspension cultivation, whereby the cells are free floating within a growth medium^14^; however, ponds and photobioreactors suffer many drawbacks such as poor CO_2_ mass transfer, high land and water usage, are vulnerable to biological contamination, and can be expensive to build and operate^15,16^. Biofilm bioreactors that circumvent suspension cultivation are more water and space efficient; however, they risk damage from desiccation, are prone to biofilm detachment (and thereby loss of the active biomass), and are no less vulnerable to biological contamination^17^.

New approaches are needed that intensify CO_2_ uptake rate and overcome the challenges that limit suspension and biofilm reactors. Photosynthetic biocomposites inspired by lichens are one such approach. Lichens are composite organisms comprising a fungus and a photobiont (microalgae and/or cyanobacteria), covering circa 12% of the Earth’s landmass^18^. The fungus provides physical support, protection and anchorage to the substratum for the photobiont, which in turn contributes carbon (as excess photosynthate) to the fungus. The proposed biocomposites are ‘lichen mimics’ wherein a concentrated population of cyanobacteria are immobilized as a thin biocoating to a supporting substratum. In addition to the cells, the biocoating comprises a polymer matrix that substitutes for the fungus. Water-based polymer emulsions or ‘latexes’ are favored as they can be biocompatible, robust, inexpensive, easy to handle, and potentially implemented on an industrial scale^19–26^.

Cell immobilization using latex polymers is greatly influenced by latex formulation and the film formation process. Emulsion polymerization is a heterogeneous process used to produce synthetic rubbers, adhesive coatings, sealants, concrete additives, paper and textile coatings, and latex paints^27^. It has several advantages over other polymerization techniques such as its high reaction rate and monomer conversion efficiency and ease of control over the products^27,28^. Monomer selection depends on the desired properties of the resulting polymeric film and with mixed monomers systems (i.e., copolymerization) the polymer properties can be altered by selection of different monomer ratios which form the resulting polymeric material^29^. Butyl acrylate and styrene are amongst the most common monomers for acrylic latexes^30^, and are used here. In addition, coalescing agents (e.g., Texanol) are typically used to promote uniform film formation, in which they can alter the properties of the polymeric latex, allowing the creation of a robust and 'continuous' (coalesced) coating. In our initial proof-of-concept study, high surface area and highly macroporous 3D biocomposites were fabricated using commercial latex-based paints applied to loofah sponge^31^. Over prolonged and continuous operation (eight weeks) the biocomposites displayed a limited capacity to retain the cyanobacteria on the loofah scaffolds due to cell outgrowth which weakened the structural integrity of the latex. In the current study, we aimed to develop a range of acrylic latex polymers of known chemical composition for continuous use for carbon capture applications that were not compromised by polymer failure. In so doing, we have demonstrated capacity to design the polymer matrix element of the lichen mimic, enabling improved biological performance with substantially enhanced mechanical resilience compared with proof-of-concept biocomposites. Further optimization will accelerate the deployment of biocomposites for carbon capture applications, particularly if paired with cyanobacteria that are metabolically engineered for enhanced CO_2_ fixation.

## Results

### Toxicity and adhesion screening

**Figure 1:**
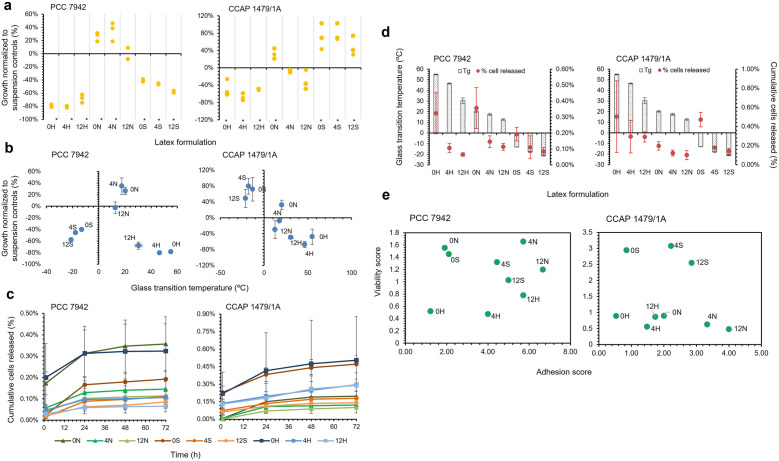
Toxicity and adhesion testing of cyanobacteria (*Synechococcus elongatus* PCC 7942 and CCAP 1479/1A) with latex formulations, relationships with glass transition temperature (Tg), and decision matrices derived from toxicity and adhesion data. (**a**) Toxicity testing using individual plots of percentage growth of cyanobacteria normalized to suspension culture controls. Treatments marked with * were significantly different from the controls. (**b**) Cyanobacteria growth data plotted against Tg of latex (Mean ± StDev; n = 3). (**c**) Cumulative number of cyanobacteria released from adhesion testing of biocomposites. (**d**) Adhesion data plotted against Tg of latexes (Mean ± StDev; n = 3). **e** Decision matrix derived from toxicity and adhesion data. ‘Hard’ (H) latex had a styrene to butyl acrylate ratio of 1:3, ‘normal’ (N) was 1:1, and ‘soft’ (S) was 3:1. The preceding number in the latex code corresponds to the Texanol content.

Nine latexes with three polymer formulations (H—‘Hard’, N—‘Normal’, S—‘Soft’) and three levels of Texanol (0, 4, 12% v/v) were tested for toxicity and adhesion with two cyanobacteria strains. Latex type significantly influenced cell growth for *S. elongatus* PCC 7942 (Scheirer-Ray-Hare test, Latex: DF = 2, H = 23.157, *P* =  < 0.001) and CCAP 1479/1A (Two-way ANOVA, Latex: DF = 2, F = 103.93, *P* =  < 0.001)  (Fig. 1a). There was no significant effect of Texanol concentration on *S. elongatus* PCC 7942 growth; only the N-latexes were non-toxic (Fig. 1a), with 0 N and 4 N supporting increased growth of 26 and 35% respectively (Mann–Whitney U, 0 N vs. 4 N: W = 13.50, *P* = 0.245; 0 N vs. control: W = 25.0, *P* = 0.061; 4 N vs. control: W = 25.0, *P* = 0.061), and 12 N sustaining growth that was comparable to the biotic controls (Mann–Whitney U, 12 N vs. control: W = 17.0, *P* = 0.885). For *S. elongatus* CCAP 1479/1A, latex blend and Texanol concentration were both significant factors, with a significant interaction between the two (Two-way ANOVA, Latex: DF = 2, F = 103.93, *P* =  < 0.001, Texanol: DF = 2, F = 5.96, *P* = 0.01, Latex*Texanol: DF = 4, F = 3.41, *P* = 0.03). The 0 N and all ‘soft’ latexes enhanced growth (Fig. 1a). There was a trend of improved growth as the styrene composition decreased.

In most cases, cell viability decreased as the Texanol concentration increased; however, there was no significant correlation for either strain (CCAP 1479/1A: DF = 25, r = − 0.208, *P* = 0.299; PCC 7942: DF = 25, r = − 0.127, *P* = 0.527). Figure 1b presents the relationship between cell growth and glass transition temperature (Tg). There were strong negative correlations between the Texanol concentration and Tg values (H-latex: DF = 7, r = − 0.989, *P* =  < 0.001; N-latex: DF = 7, r = − 0.964, *P* =  < 0.001; S-latex: DF = 7, r = -0.946, *P* =  < 0.001). The data indicate an optimal Tg for *S. elongatus* PCC 7942 growth of approximately 17 °C (Fig. 1b), whereas *S. elongatus* CCAP 1479/1A favored a Tg of below 0 °C (Fig. 1b). There was a strong negative correlation between Tg and toxicity data for *S. elongatus* CCAP 1479/1A only (DF = 25, r = − 0.857, *P* =  < 0.001).

All latexes had good adhesive affinity, with none releasing more than 1% of cells after 72 h (Fig. 1c). There were no significant differences between latexes for either *S. elongatus* strain (PCC 7942: Scheirer-Ray-Hare test, Latex * Texanol, DF = 4, H = 0.903; *P* = 0.924; CCAP 1479/1A: Scheirer-Ray-Hare test, Latex * Texanol, DF = 4, H = 3.277, *P* = 0.513). More cells were released as the Texanol concentration increased (Fig. 1c). There was a stronger negative correlation between Texanol concentration and cell adhesion affinity for *S. elongatus* CCAP 1479/1A (DF = 25, r = − 0.428, *P* = 0.026) than for *S. elongatus* PCC 7942 (DF = 25, r = − 0.660, *P* =  < 0.001)  (Fig. 1d). Furthermore, there was no statistical relationship between Tg and cell adhesion for either strain (PCC 7942: DF = 25, r = 0.301, *P* = 0.127; CCAP 1479/1A: DF = 25, r = 0.287, *P* = 0.147).

For both strains the ‘hard’ latex polymers performed poorly. In contrast, 4 N and 12 N were the best performing for *S. elongatus* PCC 7942, while 4S and 12S were best for CCAP 1479/1A (Fig. 1e); although clearly there is scope to further optimize the polymer matrix. These polymers were taken forward for semi-batch net CO_2_ absorption tests.

### Photosynthetic responses to latex polymers

**Figure 2:**
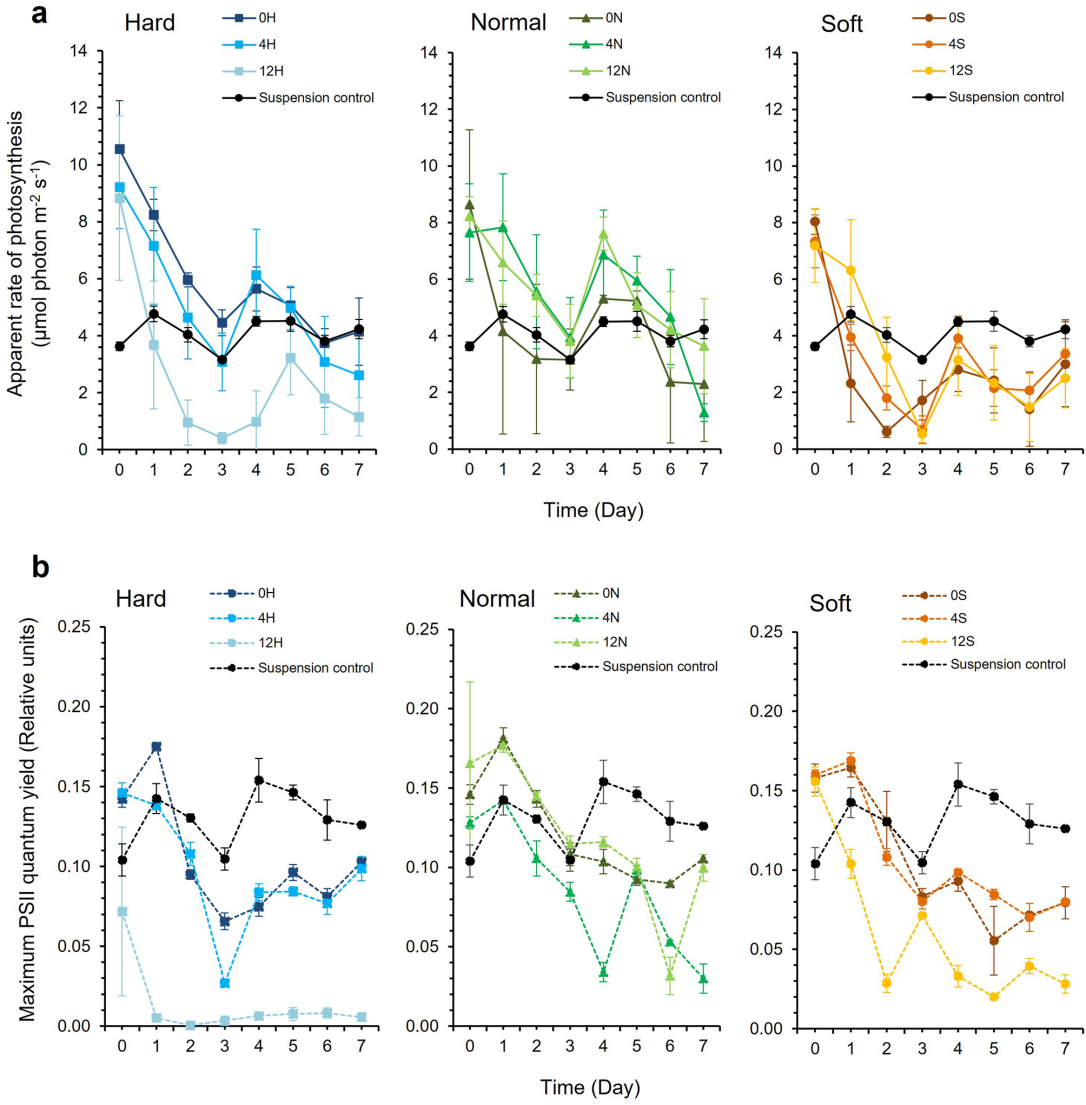
(**a**) Apparent rate of photosynthesis (PS) and (**b**) Maximum PSII quantum yield (F_v_/F_m_) of *Synechococcus elongatus* PCC 7942 in response to latex formulation compared to suspension culture controls. ‘Hard’ (H) latex had a styrene to butyl acrylate ratio of 1:3, ‘normal’ (N) was 1:1, and ‘soft’ (S) was 3:1. The preceding number in the latex code corresponds to the Texanol content. (Mean ± StDev; n = 3).

**Figure 3:**
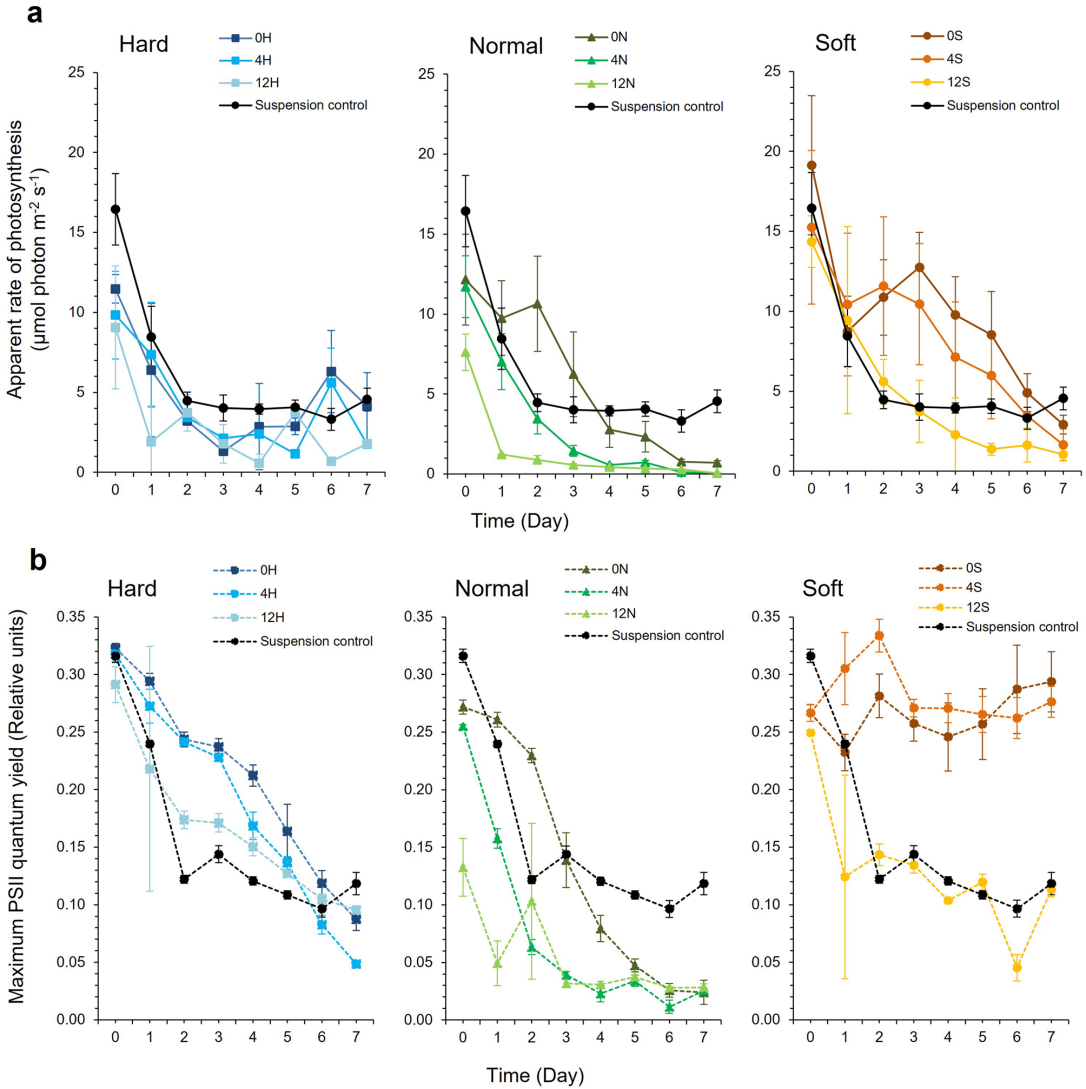
(**a**) Apparent rate of photosynthesis (PS) and (**b**) Maximum PSII quantum yield (F_v_/F_m_) of *Synechococcus elongatus* CCAP 1479/1A in response to latex formulation compared to suspension culture controls. ‘Hard’ (H) latex had a styrene to butyl acrylate ratio of 1:3, ‘normal’ (N) was 1:1, and ‘soft’ (S) was 3:1. The preceding number in the latex code corresponds to the Texanol content. (Mean ± StDev; n = 3).

Photophysiology was monitored over seven days using cells suspended in the aqueous latex formulations. In general, both the apparent rate of photosynthesis (PS) and the maximum PSII quantum yield (F_v_/F_m_) decreased over time, although the decreases were not uniform and several of the PS datasets displayed a bi-phasic response indicating partial, albeit short-lived recovery of PS activity (Figs. 2a and 3b). The biphasic response was less pronounced for F_v_/F_m_ (Figs. 2b and 3b).

For *S. elongatus* PCC 7942, latex formulation and Texanol concentration did not interact to affect PS over time (GLM, Latex * Texanol * Time, DF = 28, F = 1.49, *P* = 0.07), although the formulation was a significant factor (GLM, Latex * Time, DF = 14, F = 3.14, *P* =  < 0.001)  (Fig. 2a). Texanol concentration did not have a significant effect over time (GLM, Texanol * Time, DF = 14, F = 1.63, *P* = 0.078). There were significant interactions affecting F_v_/F_m_ (GLM, Latex * Texanol * Time, DF = 28, F = 4.54, *P* =  < 0.001). An interaction between latex formulation and Texanol concentration had a significant effect on F_v_/F_m_ (GLM, Latex * Texanol, DF = 4, F = 180.42, *P* =  < 0.001). Each parameter also influenced F_v_/F_m_ over time (GLM, Latex * Time, DF = 14, F = 9.91, *P* =  < 0.001 and Texanol * Time, DF = 14, F = 10.71, *P* =  < 0.001). The 12H latex supported the lowest mean PS and F_v_/F_m_ values (Fig. 2b), indicating that the polymer was more toxic.

There were significant differences in PS for *S. elongatus* CCAP 1479/1A (GLM, Latex * Texanol * Time, DF = 28, F = 2.75, *P* =  < 0.001), with latex formulation but not Texanol concentration as significant factors (GLM, Latex * Time, DF = 14; F = 6.38; *P* =  < 0.001; GLM, Texanol * Time, DF = 14, F = 1.26, *P* = 0.239). The 0S and 4S ‘soft’ polymers supported slightly higher PS performance levels than the suspension controls (Mann–Whitney U, 0S vs. control, W = 686.0, *P* = 0.044, 4S vs. control, W = 713, *P* = 0.01) and supported improved F_v_/F_m_ performance (Mann–Whitney U, 0S vs. control, W = 794.0, *P* =  < 0.001, Mann–Whitney U, 4S vs. control, W = 815.0, *P* =  < 0.001)  (Fig. 3a) indicating more efficient photon transport into photosystem II. For the F_v_/F_m_ values of CCAP 1479/1A cells, there were significant differences between latexes over time (GLM, Latex * Texanol * Time, DF = 28, F = 6.00, *P* =  < 0.001)  (Fig. 3b).

**Figure 4:**
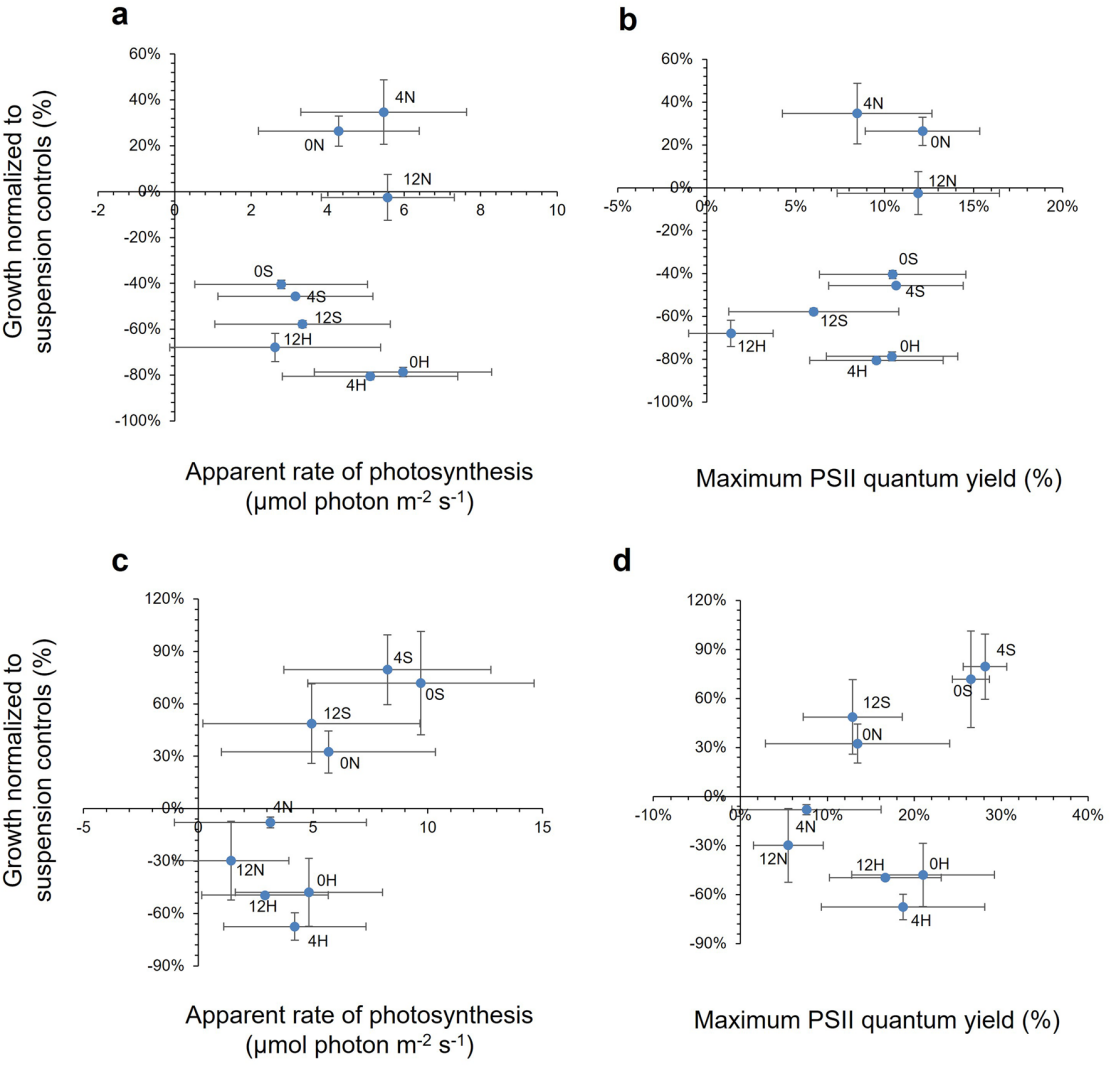
The relationship between growth and photophysiology for *Synechococcus elongatus* in response to latex formulation. (**a**) Toxicity data plotted against the apparent rate of photosynthesis (PS) and (**b**) the maximum PSII quantum yield (F_v_/F_m_) of PCC 7942. **c** Toxicity data plotted against PS and **d** F_v_/F_m_ of CCAP 1479/1A. ‘Hard’ (H) latex had a styrene to butyl acrylate ratio of 1:3, ‘normal’ (N) was 1:1, and ‘soft’ (S) was 3:1. The preceding number in the latex code corresponds to the Texanol content. (Mean ± StDev; n = 3).

Figure 4 plots the mean PS and F_v_/F_m_ values over the seven days period against cell growth for each strain. There were no clear patterns for *S. elongatus* PCC 7942 (Fig. 4a and b); however, CCAP 1479/1A indicated a parabolic relationship for PS (Fig. 4c) and F_v_/F_m_ values (Fig. 4d) with growth broadly following the change in the styrene and butyl acrylate ratio.

### CO_2_ fixation rates

**Figure 5:**
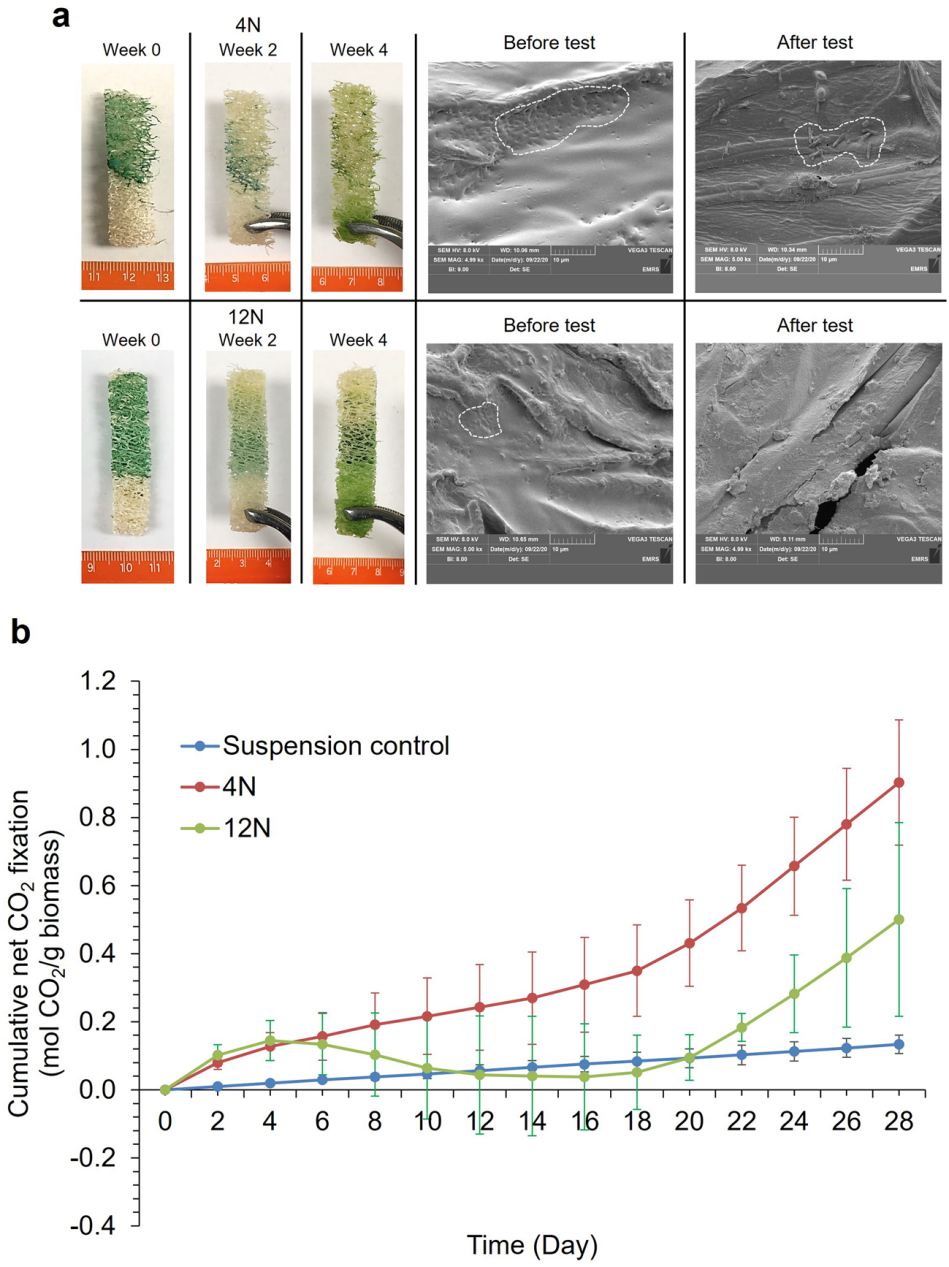
Semi-batch CO_2_ absorption tests with *Synechococcus* elongatus PCC 7942 biocomposites with the 4 N and 12 N latexes. (**a**) Images demonstrate cell release and pigment bleaching as well as SEM images of biocomposites before and after tests. White dash lines indicate where cells were deposited on the biocomposites. (**b**) cumulative net CO_2_ absorption over the four weeks period. ‘Normal’ (N) latex had a styrene to butyl acrylate ratio of 1:1. The preceding number in the latex code corresponds to the Texanol content. (Mean ± StDev; n = 3).

The PCC 7942 biocomposites had limited effectiveness in retaining cells, with considerable cell leaching within the first four weeks (Fig. 5). After an initial CO_2_ absorption phase, cells immobilized with the 12 N latex began to release CO_2_, maintaining this pattern between days 4 to 14 (Fig. 5b). These data correspond to observations of pigment bleaching. Net CO_2_ absorption commenced again from day 18. Despite the release of cells (Fig. 5a), the PCC 7942 12 N biocomposites still accumulated more CO_2_ than the suspension controls across the 28 days, although not significantly so (Mann–Whitney U test, W = 2275.5; *P* = 0.066). The CO_2_ absorption rates with the 12 N and 4 N latexes were 0.51 ± 0.34 and 1.18 ± 0.29 g CO_2_ g^−1^_biomass_ d^−1^. There were statistically significant differences between treatment and time levels (Scheirer-Ray-Hare test, Treatment: DF = 2, H = 70.62, *P* =  < 0.001 Time: DF = 13, H = 23.63, *P* = 0.034) but there was no significant interaction between treatment and time (Scheirer-Ray-Hare test, Time * Treatment: DF = 26, H = 8.70, *P* = 0.999).

**Figure 6:**
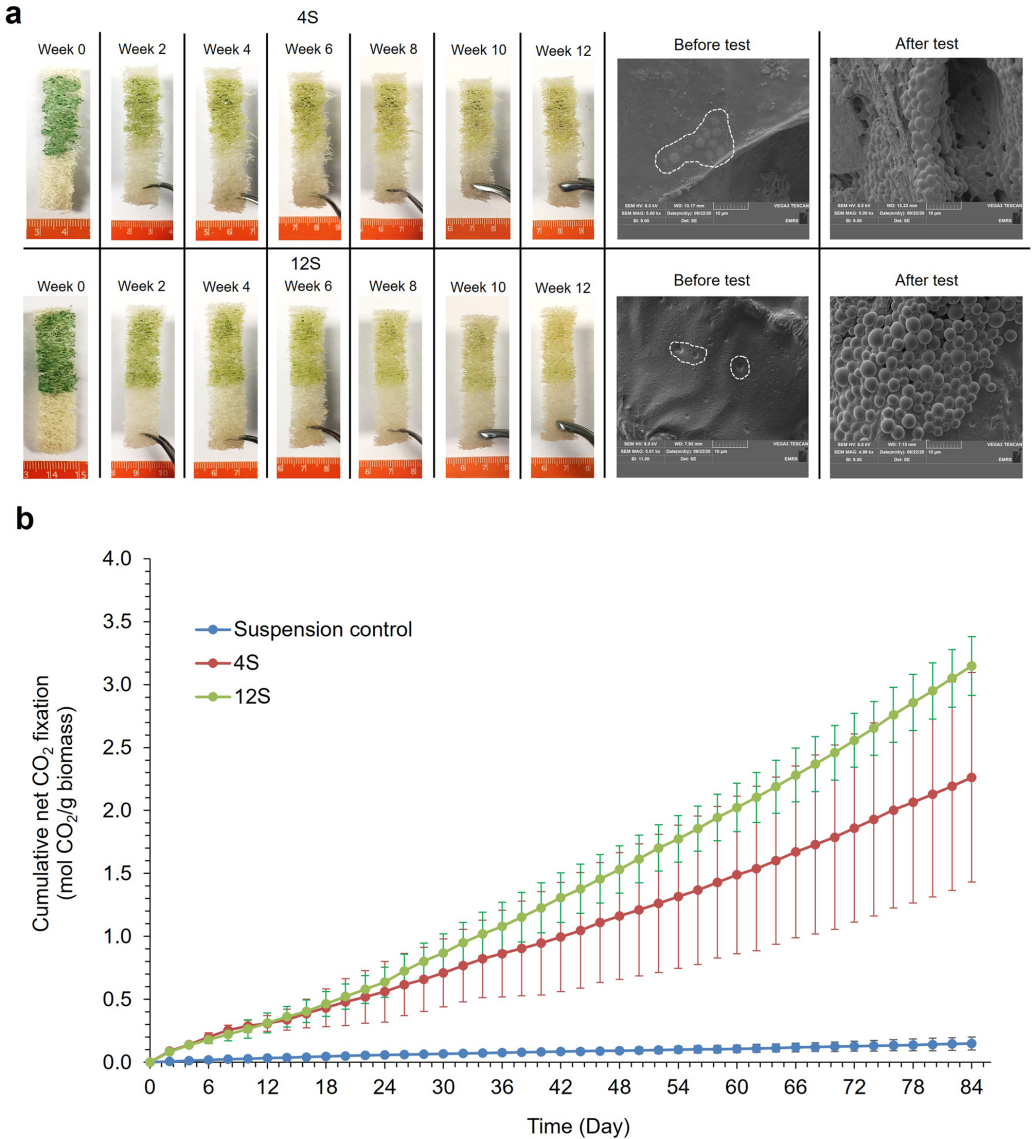
Semi-batch CO_2_ absorption tests with *Synechococcus* elongatus CCAP 1479/1A biocomposites with the 4 N and 12 N latexes. (**a**) Images demonstrate cell release and pigment bleaching as well as SEM images of biocomposites before and after tests. White dash lines indicate where cells were deposited on the biocomposites. (**b**) cumulative net CO_2_ absorption over the twelve weeks period. ‘Soft’ (S) latex had a styrene to butyl acrylate ratio of 1:1. The preceding number in the latex code corresponds to the Texanol content. (Mean ± StDev; n = 3).

Cell retention was much improved for the CCAP 1479/1A strain with 4S and 12S, despite the pigments slowly discoloring with time (Fig. 6a). CCAP 1479/1A biocomposites absorbed CO_2_ for the full 84 days (12 weeks) without additional nutrient supplementation. SEM analysis (Fig. 6a) supported the visual observation of little cell detachment. Initially, the cells were embedded within the latex coatings and, despite cell growth, the integrity held. The CO_2_ absorption rates were significantly higher than the suspension controls (Scheirer-Ray-Hare test, Treatment: DF = 2; H = 240.59; *P* =  < 0.001, Time: DF = 42; H = 112; *P* =  < 0.001)  (Fig. 6b). The 12S biocomposites achieved the highest CO_2_ absorption rate (1.57 ± 0.08 g CO_2_ g^−1^_biomass_ d^−1^), with the 4S latex being 1.13 ± 0.41 g CO_2_ g^−1^_biomass_ d^−1^, but they were not significantly different (Mann–Whitney U test, W = 1507.50; *P* = 0.07) and there was no significant interaction between treatment and time (Scheirer-Ray-Hare test, Time * Treatment: DF = 82; H = 10.37; *P* = 1.000).

There were no significant differences in carbohydrate content for either *S. elongatus* PCC 7942 (Scheirer-Ray-Hare test, Time * Treatment: DF = 4, H = 3.243, *P* = 0.518) or *S. elongatus* CCAP 1479/1A biocomposites (two-way ANOVA, Time * Treatment: DF = 8, F = 1.79, *P* = 0.119) (Fig. S4). Carbohydrate content was highest by week 2 in the PCC 7942 biocomposites (4 N = 59.4 ± 22.5% w/w, 12 N = 67.9 ± 3.3% w/w), whereas the carbohydrate content of the suspension control was highest by week 4 (Control = 59.6 ± 2.84% w/w). Apart from at the start of the trial, the total carbohydrate content of the CCAP 1479/1A biocomposites was equivalent to the suspension controls, with some variation in the 12S latex on week 4. The highest biocomposite values were 51.9 ± 9.6% w/w for 4S and 77.1 ± 17.0% w/w for 12S.

## Discussion

We set out to demonstrate capacity to design for improved structural integrity of thin film latex polymer coatings as an essential component of a lichen-mimic biocomposite concept, without compromising on biocompatibility or operational performance. Indeed, if the structural problems associated with cell outgrowth were overcome we would anticipate substantial performance improvements beyond our proof-of-concept biocomposites which were already rivalling other cyanobacteria and microalgae carbon capture systems^31^.

Coatings must be non-toxic, robust, support long-term cell adhesion, and should be porous to facilitate effective CO_2_ mass transfer and O_2_ off-gassing. Acrylic latex type polymers are easily formulated and widely used in the coatings, textiles and adhesives industries^30^. We combined cyanobacteria with water-borne acrylic latex polymer emulsions polymerized from defined ratios of styrene/butyl acrylate particles and with differing Texanol concentrations. Styrene and butyl acrylate were selected to enable manipulation of physical properties, particularly the elasticity of the coating and coalescing efficiency (essential for a robust and high adhesion coating), allowing synthesis of ‘hard’ and ‘soft’ polymer particles. The toxicity data indicated that ‘hard’ latexes with a high styrene content were unfavorable for cyanobacteria survival. Unlike butyl acrylate, styrene is recognized as toxic to algae^32,33^. The cyanobacteria strains responded quite differently to the latexes, identifying an optimal glass transition temperature (Tg) for *S. elongatus* PCC 7942, whereas *S. elongatus* CCAP 1479/1A had a negative linear relationship with Tg.

Drying temperature affects the capacity to form a continuous homogeneous latex film. If the drying temperature is below the minimum film formation temperature (MFFT), the polymeric latex particles will not fully coalesce, resulting in adhesion at the particle–particle interface only. The resulting film can suffer from weak cohesive and mechanical strength and may even take on a powdery form^29^. The MFFT is closely related to Tg and can be controlled by monomer composition and through the inclusion of coalescing agents, such as Texanol. The Tg defines many of the physical properties of the resulting coating, which can occupy either a rubbery or glassy state^34^. Tg is influenced by monomer type and relative percentage composition according to the Flory-Fox Equaion^35^. Adding a coalescing agent can lower the MFFT through non-permanent suppression of the latex particle Tg, enabling film formation at lower temperatures, but still allowing formation of a hard and robust coating, as the coalescing agent slowly evaporates or is extracted over time^36^.

Increasing Texanol concentration promoted film formation by softening the polymer particles (lowering the Tg) by absorption into the particles during drying^37^, improving cohesive film strength and cell adhesion. As the biocomposites were dried at ambient temperature (∼ 18–20 °C), the Tg of the ‘hard’ latexes (30 to 55 °C) were higher than the drying temperature, meaning that particle coalescence may be sub-optimal, resulting in film remaining in the glassy state with poor mechanical and adhesive properties, with limited elasticity and diffusion rates^30^, ultimately resulting in greater cell loss. Film formation in the ‘normal’ and ‘soft’ polymers occurred at or below the polymer film Tg, with film formation benefiting from improved coalescence, resulting in continuous polymeric films with improved mechanical, cohesive and adhesive properties. The resulting films would remain in the rubbery state during the CO_2_ capture experiments as their Tg was close to (‘normal’ blend: 12 to 20 ºC) or well below (‘soft’ blend: − 21 to − 13 °C) the ambient temperature^30^. ‘Hard’ latexes (3.4 to 2.9 kgf mm^−1^) were three times harder than the ‘normal’ blends (1.0 to 0.9 kgf mm^−1^). The hardness of the ‘soft’ latexes could not be measured using microhardness due to their excessive rubbery behavior and tackiness at room temperature. The surface charge is also likely to influence the adhesion affinity, but further data are needed to provide meaningful insights. Nevertheless, all of the latexes were effective at retaining the cells, releasing less than 1%.

Photosynthetic performance decreased over time. Exposure to polystyrene can cause membrane destruction and oxidative stress^38–41^. The F_v_/F_m_ values from *S. elongatus* CCAP 1479/1A exposed to 0S and 4S were enhanced almost two-fold compared to suspension controls, which corresponded well with the 4S biocomposite CO_2_ absorption rates, irrespective of the low average PS values. The higher F_v_/F_m_ values infer that the electron transport to PSII can deliver more photons^42^ potentially leading to higher CO_2_ fixation rates. However, it should be noted that the photophysiology data were obtained from cells suspended in the aqueous latex solution and may not necessarily be directly comparable with the mature biocomposites.

There could be the potential for cellular stress and reduced performance if the latexes presented barriers to light and/or gas exchange, causing light and CO_2_ limitation, and photorespiration should O_2_ off-gassing be compromised^39^. Light transmission through cured coatings was assessed, with the ‘hard’ latexes showing minor transmission losses between 440 and 480 nm (partially ameliorated by increasing the Texanol concentration through improved film coalescence), whereas the ‘soft’ and ‘normal’ latexes showed no significant losses. The analyses, as well as all incubations, were conducted at low light intensities (30.5 μmol m^−2^ s^−1^); therefore, any loss in photosynthetically active radiation attributable to the polymer matrix would be more than compensated at irradiances more typical of daylight or intensive artificial illumination, and may even be beneficial in preventing photoinhibition at damaging light intensities.

The CCAP 1479/1A biocomposites functioned for the 84 days of this trial without nutrient refreshment or noticeable biomass loss—a key objective of the study. The depigmentation of the cells was likely due to a chlorosis process that acted as a response to nitrogen starvation to enable long term survival (dormant-like state), which may assist the cells to reinitiate growth once sufficient nitrogen accumulation is reached^43^. The SEM images confirmed that the cells were retained within the coatings despite cell division having occurred, evidencing the elasticity of the ‘soft’ latexes, thus demonstrating a clear advance beyond the proof-of-concept versions. The ‘soft’ latexes were around 70% butyl acrylate by weight, which is well above the concentration reported to produce flexible coatings after drying^44^.

The net CO_2_ absorption rates were substantially higher than the suspension controls (by 14–20 and 3–8 times for *S. elongatus* CCAP 1479/1A and PCC 7942 respectively). Previously, using a CO_2_ mass transfer model, we showed that the main driving force for high CO_2_ uptake was a steep CO_2_ concentration gradient at the biocomposite surface^31^, with biocomposite performance potentially limited by mass transfer resistance. This could be addressed by introducing non-toxic, non-film forming components into the latex formulations to increase coating porosity and permeability^26^, but may compromise cell retention as this strategy will inevitably lead to weaker films^20^. It is possible to alter chemical composition to increase porosity during polymerization, which is a better option particularly from industrial and scalability perspectives^45^.

The performance of the new biocomposites compared favorably with recent studies using microalgae and cyanobacteria biocomposites when adjusted for cell loading rate (Table 1)^21,46^, and were operated over much longer periods (84 days versus 15 h^46^ and 3 weeks^21^).

**Table 1:** Comparison of the highest CO_2_ absorption rates with biocomposites relative to selected literature values.

Species and strain	Type	System description	CO_2_ fixation rate		Ref
			(mmol CO_2_ m^−2^ d^−1^)	(g CO_2_ g^−1^_biomass_ d^−1^)	
*Synechococcus* PCC 7002	Cyanophyte	Paper-based biocomposites with 20% v/v CO_2_ at 25 °C; light: 100 µmol m^−2^ s^−1^ in batch for 500 h	136	0.22	^21^
*Chlorella vulgaris*	Freshwater chlorophyte	Biopolymer porous paper pulp in microfiber cellulose mixed with chitosan matrix in BG11 and fixed onto spinning disk reactor for 15 h at 300 rpm with 5% v/v CO_2_	110.64	1.38	^46^
*Chlorella vulgaris*	Freshwater chlorophyte	Immobilized to loofah at 18 °C; light: 16:8 photoperiod at 30.5 µmol m^−2^ s^−1^ with continuous 5% v/v CO_2_ at day 42	2.38	0.17	^31^
*Dunaliella salina*	Marine chlorophyte	Immobilized to loofah at 18 °C; light: 16:8 photoperiod at 30.5 µmol m^-2^ s^-1^ with continuous 5% v/v CO_2_ at day 42	3.44	0.25	^31^
*S. elongatus* CCAP 1479/1A	Cyanophyte	Immobilized to loofah at 18 °C; light: 16:8 photoperiod at 30.5 µmol m^−2^ s^−1^ with 5% v/v CO_2_ in semi-batch at day 56	6.15	0.93	^31^
*S. elongatus* CCAP 1479/1A	Cyanophyte	Immobilized to loofah with 12S latex at 18 °C; light: 16:8 photoperiod at 30.5 µmol m^−2^ s^−1^ with 5% v/v CO_2_ in semi-batch at day 84	9.57	1.57	This study

The bulk cellular carbohydrate content compared favorably with other studies using cyanobacteria^47–50^, and was used as a potential yardstick for carbon capture and utilization/recycling applications, e.g., for fermentation processes^49,51^ within a BECCS framework or for the manufacture of biodegradable bioplastics^52^. As part of the rationale for this study, we suggested that afforestation, even if considered within a negative emissions BECCS concept, was not the panacea for climate change, and would consume an alarming proportion of global arable land^6^. As a thought experiment, it has been estimated that between 640 and 950 GtCO_2_ must be removed from the atmosphere by 2100 to limit global temperature rise to 1.5 °C^53^ (approximating to 8 to 12 GtCO_2_ per annum). To achieve this using the best performing biocomposite (574.08 ± 30.19 tCO_2_ t^−1^_biomass_ yr^−1^) would require scaling to 5.5 × 10^10^ to 8.2 × 10^10^ m^3^ (assuming the equivalent photosynthetic efficiency), containing between 196 and 292 million litres of polymer. Assume that 1 m^3^ of biocomposite occupies a land footprint of 1 m^2^, the area required to absorb the target total annual global CO_2_ would range between 5.5 and 8.17 million hectares; equivalent to 0.18–0.27% of habitable land in the tropics, and reducing the land requirement for BECCS by 98–99%. It should be noted that the theoretical scaled capture was based on the CO_2_ absorption rates recorded under low light exposure. Once the biocomposites are exposed to natural light, which is of higher intensity, the CO_2_ uptake rates should increase, which will further decrease the land requirement and tip the balance further towards the biocomposites concept. However, implementation location would need to be equatorial to obtain consistently high light intensity and duration.

The global CO_2_ fertilization effect—i.e., the enhancement in vegetation productivity driven by increased CO_2_ availability—has decreased in most terrestrial regions, likely due to changes in key soil nutrients (N and P) and water resources^7^. This means that terrestrial photosynthesis may not result in increased CO_2_ uptake, despite rising CO_2_ concentrations in air. With this scenario, land-based climate mitigation strategies such as BECCS are even less likely to succeed. If this global phenomenon holds true, our lichen inspired biocomposites could become a pivotal asset by turning unicellular aquatic photosynthetic microorganisms into ‘terrestrial proxies’. Most terrestrial plants fix CO_2_ through C3 photosynthesis, with C4 plants favored in warmer and drier habitats and proving more efficient at higher CO_2_ partial pressures^54^. Cyanobacteria offer an alternative that could negate the worrying projections for the impact of a weakening CO_2_ fertilization effect on C3 plants. Cyanobacteria have overcome photorespiratory limitations by evolving efficient carbon concentrating mechanisms^55^, wherein higher partial pressures of CO_2_ are presented to, and maintained around ribulose-1,5-bisphosphate carboxylase/oxygenase (RuBisCo) within carboxysomes. If production of cyanobacteria biocomposites can be scaled, this could represent an important weapon in humanities’ arsenal against climate change.

Biocomposites (lichen mimics) hold distinct advantages over conventional microalgae and cyanobacteria suspension-based cultures by providing higher CO_2_ uptake rates, minimizing the risk of contamination, and promising much lower land, water and nutrients usage at a competitive CO_2_ avoidance cost^56^. This study has demonstrated the capacity to design and manufacture high performance biocompatible latex that, when paired with loofah sponge as a candidate scaffold, deliver effective and efficient CO_2_ absorption over months-long operational periods with minimal cell loss. The biocomposites could theoretically capture around 570 tCO_2_ t^−1^_biomass_ yr^−1^, and could prove to be more crucial in our fight against climate change than an afforestation-BECCS strategy. With further optimization of the polymer formulation, testing under higher light intensities and, if married with carefully considered metabolic engineering, nature’s original bio-geoengineers may once again ride to the rescue.

## Methods

### Latex polymer emulsions

Acrylic latex polymers were prepared using styrene, butyl acrylate and acrylic acid monomer mixtures, adjusted to pH 7 using 0.1 M sodium hydroxide (Table 2). Styrene and butyl acrylate comprised the bulk of the polymer chain, while acrylic acid helped maintain the latex particles in suspension^57^. The structural performance of the latex was defined by the glass transition temperature (Tg) which was controlled by altering the ratio of styrene to butyl acrylate, offering ‘hard’ and ‘soft’ characteristics respectively^58^. A typical acrylic latex polymer consists of 50:50 styrene: butyl acrylate^30^; hence, in this study latexes with that ratio were termed ‘normal’, those with more styrene were termed ‘hard’ and those with less styrene were termed ‘soft’.

**Table 2:** Latex polymer composition, glass transition temperature and bulk properties.

Type of latex	Latex code	Texanol (% v/v)	Glass transition temperature (°C)	Styrene (g; mol)	Butyl acrylate (g; mol)	Acrylic acid (g; mol)	% solid content (w/w)
Hard	0H	0	55.1 ± 0.5	375; 3.6	120; 0.94	10; 0.14	40.9 ± 0.4
	4H	4	46.5 ± 0.5	375; 3.6	120; 0.94	10; 0.14	42.2 ± 0.3
	12H	12	30.4 ± 2.6	375; 3.6	120; 0.94	10; 0.14	43.7 ± 0.6
Normal	0 N	0	20.3 ± 0.8	250; 2.4	240; 1.87	10; 0.14	39.3 ± 0.3
	4 N	4	17.6 ± 0.9	250; 2.4	240; 1.87	10; 0.14	39.9 ± 0.4
	12 N	12	12.6 ± 0.8	250; 2.4	240; 1.87	10; 0.14	40.7 ± 0.2
Soft	0S	0	− 13.1 ± 0.2	125; 1.2	360; 2.81	10; 0.14	41.8 ± 0.1
	4S	4	− 17.9 ± 0.7	125; 1.2	360; 2.81	10; 0.14	42.3 ± 0.2
	12S	12	− 21.1 ± 0.5	125; 1.2	360; 2.81	10; 0.14	44.8 ± 0.7

‘Hard’ (H) latex had a styrene to butyl acrylate ratio of 1:3, ‘normal’ (N) was 1:1, and ‘soft’ (S) was 3:1. The preceding number in the latex code corresponds to the Texanol content.

A primary emulsion was made using distilled water (174 g), sodium bicarbonate (0.5 g), and a surfactant Rhodapex Ab/20 (30.92 g) (Solvay) to stabilize the monomer droplets^30^. A secondary aliquot, comprising of styrene, butyl acrylate, and acrylic acid as specified in Table 2, was added dropwise to the primary emulsion over 4 h at 100 mL h^−1^ using a glass syringe (Science Glass Engineering) assisted with a syringe pump (Cole-Palmer, Vernon Hills, Illinois). An initiator solution to promote polymerization^59^ was prepared using dH_2_O and ammonium persulfate (100 mL, 3% w/w).

A solution containing dH_2_O (206 g), sodium bicarbonate (1 g), and Rhodapex Ab/20 (4.42 g) was stirred with a stainless-steel propeller using an overhead stirrer (Heidolph Hei-TORQUE value 100) and heated to 82 °C in a water jacketed vessel attached to a VWR Scientific 1137P heating water bath. A reduced mass of monomer (28.21 g) and initiator (20.60 g) solutions were added dropwise to the jacket vessel and stirred for 20 min. The remaining monomer (150 mL h^−1^) and initiator (27 mL h^−1^) solutions were stirred vigorously to maintain particle suspension until they were added to a water jacketed vessel over 5 h using 10 mL and 100 mL syringes respectively assisted with a syringe pump. Stirrer speeds were increased in relation to the increasing suspension volume to ensure the suspension was retained. The reaction temperature was increased to 85 °C once both initiator and emulsion solutions had been added, mixed thoroughly for 30 min at 450 RPM, and then cooled to 65 °C. Two chaser solutions were added to the latex once cooled; tert-butyl hydroperoxide (t-BHP) (70% in water) (5 g, 14% w/w) and isoascorbic acid (5 g, 10% w/w). t-BHP was added dropwise and left to react for 20 min. Isoascorbic acid was then added at 4 mL h^-1^ from a 10 mL syringe using a syringe pump. The latex solution was then allowed to cool to room temperature and adjusted to pH 7 with 0.1 M sodium hydroxide.

2,2,4-Trimethyl-1,3-pentanediol monoisobutyrate (Texanol)—a low toxicity biodegradable coalescence agent used in latex paints^37,60^—was added in three volumes using a syringe and pump (0, 4, 12% v/v) as a coalescence agent to the latex mixture to promote film formation during drying^37^. The percentage solids content of the latexes was determined by oven drying 100 µL of each polymer in pre-weighed aluminum foil caps for 24 h at 100 °C.

### Polymer matrix characterization

For light transmission, each latex blend was coated onto glass microscope slides using a stainless-steel draw-down cube calibrated to prepare 100 µm thickness films and dried at 20 °C for 48 h. Light transmission (focusing on photosynthetically active radiation; *λ* 400–700 nm) was measured using an ILT950 SpectriLight Spectroradiometer, with the sensor 35 cm from 30 W daylight-type fluorescent tubes (Sylvania Luxline Plus, n = 6)—the light source under which the cyanobacteria and biocomposites were maintained. SpectrILight III version 3.5 software was used to record irradiance and transmission at λ 400–700 nm^61^. All samples were placed on top of the sensor and an uncoated microscope slide was used as a control.

Prior to hardness testing, latex samples were added to a silicone baking tray and allowed to dry for 24 h. Dried latex samples were placed onto a steel mantle under a × 10 microscope lens. Once in focus the sample was assessed using a Buehler Micromet II microhardness tester. Samples were subjected to forces ranging from 100 to 200 g with a set 7 s of loading time, creating a diamond indentation within the sample. This indent was analyzed using a Bruker Alicona × 10 microscope lens with the additional shape measuring software. Vickers Hardness formula (Eq. 1) was used to calculate the hardness of each latex; where, *HV* is the Vickers number, *F* is the applied force, and *d* is the mean value of the diagonals of the indentation calculated from the height and width of the indent. ‘Soft’ latexes were unable to be measured due to sticking and stretching during indentation testing.

1 $$HV = \frac{{2Fsin \left( {\frac{136^\circ }{2}} \right)}}{{d^{2} }}$$

To determine, glass transition temperature (Tg) of the latex formulation, polymer samples were deposited into a silicone tray, dried for 24 h, weighed to 0.005 g, and placed into a sample pan. The pan was covered and placed into a differential scanning colorimeter (PerkinElmer DSC 8500, Intercooler II, Pyris Data Analysis software)^62^. The heat flux method was used to situate both reference and sample pans within the same furnace with integrated temperature sensors for temperature measurement. A total number of two ramps were used to create a consistent curve. Sample methods performed a repeated ramp from − 20 °C to 180 °C at a rate of 20 °C per min. Each beginning and end point was held for 1 min to account for temperature lag.

### Semi-batch CO_2_ absorption test

To evaluate the biocomposite’s CO_2_ uptake performance, the samples were prepared and tested in the same manner as our previous study^31^. Dried and autoclaved loofah were cut into strips of approximately 1 × 1 × 5 cm and weighed. Six hundred microlitres of the two best performing biocoatings for each cyanobacteria strain were pipetted onto one end of each loofah strip, covering approximately 1 × 1 × 3 cm and dried at 20 °C for 24 h in darkness. Due to the macroporous structure of the loofah, some of the formulation was lost as waste; hence cell loading efficiency was not 100%. To overcome this, the mass of dried formulation on the loofah was determined and normalized with a reference dried formulation. Abiotic controls were similarly prepared, consisting of loofah, latex and sterile growth medium.

To set up the semi-batch CO_2_ absorption tests, the biocomposites were placed (n = 3) into 50 mL glass bottles with one end of the biocomposite (without biocoatings) contacted with 5 mL of growth medium allowing nutrient delivery via capillary action. The bottles were sealed with 20 mm butyl rubber stoppers and crimped using silver aluminium caps. After sealing, 45 mL of 5% CO_2_/air gas mixture was injected with a sterile needle attached to an air-tight syringe. Suspension controls (n = 3) were established with a cell density equivalent to the cell loading within the biocomposite in the growth medium. The tests were conducted at 18 ± 2 °C with a 16:8 light:dark photoperiod at 30.5 μmol m^−2^ s^−1^. The headspace was removed every two days with an air-tight syringe and analyzed using a GEOTech G100 infrared absorption CO_2_ meter to determine the percentage of CO_2_ absorbed. The equivalent volume of CO_2_ gas mixture was replenished.

The % CO_2_ fixed was calculated as: %CO_2_ fixed = 5% (v/v)—%CO_2_ recorded (Eq. 2), where *P* = pressure, *V* = volume, *T* = temperature and *R* = ideal gas constant.

2 $$net \,CO_{2} \,absorption\, rate \left( {mol\, CO_{2} g_{biomass}^{ - 1} day^{ - 1} } \right) = \frac{{\% CO_{2} \,fixed \times \frac{PV}{{RT}}}}{Immobilised \,dried \,biomass \left( g \right) \times 2 \,days}$$

The CO_2_ absorption rates reported for the cyanobacteria suspension controls and the biocomposites were normalized against the abiotic controls. The functional unit of g biomass is the amount of dried biomass immobilized onto the loofah. It was determined by weighing the loofah sample before and after cell immobilization. The mass of the cell loading (which is equivalent to biomass) was considered by separately weighing the formulation before and after drying and by calculating the density of the cell formulation (Eq. 3). It was assumed that the cell formulation was uniform during the immobilization.

3 $$Immobilized\, dried\, biomass = cell\, loading\, \left( \% \right) \times volume \,of\, cell \,formulation \,\left( \frac{g}{mL} \right) \times density \,of \,cell\, formulation\, \left( \frac{g}{mL} \right)$$

### Statistical analysis

Minitab 18 and Microsoft Excel with RealStatistics Add-in were used for statistical analyses. Normality was tested with the Anderson–Darling test, and equality of variance tested using the Levene’s test. Data that met these assumptions were analyzed using two-way analysis of variance (ANOVA) with Tukey’s test as a post-hoc analysis. Two factor data that did not meet the assumptions of normality and equality of variance were analyzed using the Scheirer-Ray-Hare test, followed by the Mann–Whitney U test to identify significance between treatments. Generalized Linear Mixed (GLM) models were used for non-normal data with three factors, in which the data were transformed using a Johnson transformation^63^. Pearson’s product-moment correlations were conducted to assess relationships between Texanol concentration, glass transition temperature, and latex toxicity and adhesion data.

## Supporting information

The content supplied in the supporting information file consists of methods of cyanobacteria cultivation, toxicity and cell adhesion tests, photosynthesis analysis, loofah preparation, biocomposite microstructure analysis and total carbohydrate extraction. Figures S1, S2, and S3 represent results of latex characterization and Fig. S4 is the total carbohydrate content extracted from *Synechococcus elongatus* biocomposites during the semi-batch CO_2_ absorption tests compared with suspension culture controls.

## Supplementary Information

Supplementary Information.

## Data Availability

The data that support the findings of this study are available from the corresponding authors upon reasonable request.
