# Supplementary material for: Engineered living photosynthetic biocomposites for intensified biological carbon capture
Source: Sci Rep. 2022 Nov 4;12:18735. doi: 10.1038/s41598-022-21686-3 (PMC9636219; doi:10.1038/s41598-022-21686-3)
Supplement: Supplementary file 1 — Supplementary Information. [file 41598_2022_21686_MOESM1_ESM.docx]

**Engineered living photosynthetic biocomposites for intensified biological carbon capture**

Pichaya In-na^1,7^, Elliot B. Sharp^2^, Gary S. Caldwell^3^*, Matthew G. Unthank^4^, Justin J. Perry^5^*, Jonathan G.M. Lee^6^*

SUPPORTING INFORMATION

**Loofah preparation.** Pretrimmed sections of loofah (aka luffa) were purchased from retail outlets. They were washed repeatedly with sterile deionized water (dH_2_O), autoclaved at 125 °C for 15 mins, dried at 105 °C for 3 h, and kept in a silica gel desiccator until use.

**Cyanobacteria cultivation.** Two freshwater strains of *Synechococcus elongatus* were grown; *S. elongatus* CCAP 1479/1A was cultivated in JM medium without cyanocobalamin, thiamine HCl, and biotin, while *S. elongatus* PCC 7942 was cultivated in BG11 medium. All nutrient chemicals were purchased from Sigma Aldrich UK. The cultures were grown in 10L polycarbonate carboys (Nalgene) maintained at 18 ± 2 °C with a 16:8 h light:dark photoperiod at 30.5 μmol m^-2^ s^-1^ from 30W daylight-type fluorescent tubes (Sylvania Luxline Plus, n = 6). Mixing and gas exchange were through constant HEPA filtered aeration (normal atmospheric composition) using an aquarium pump (KOI AIR 50 Blagdon).

**Toxicity and cell adhesion tests.** Contact toxicity and adhesion tests followed the procedure outlined in our previous study ^1^. Briefly, 1 mL of cyanobacteria culture (12-14 days old) was combined with 1 mL of 5% w/w latex emulsion in 24-well multiplates. The samples were mixed by daily forward and reverse pipetting over seven days and incubated at 20 °C, with a 16:8 light:dark photoperiod at 30.5 μmol m^-2^ s^-1^. Cell counts were performed at the end of the tests using an improved Neubauer Hawksley hemocytometer and scored relative to suspension controls.

For adhesion tests, 2 L of cyanobacteria culture were centrifuged for 30 mins at 1717 RCF (Sigma Laboratory Centrifuges, 3K18 C) to obtain a wet cell paste. The autoclaved and dried loofah were cut into approximately 1 $\times$ 1 $\times$ 1 cm cubes and placed into 24-well multiplates. One mL of latex (5.0% w/w) was mixed for 10 to 20 seconds by vortex with 25 µL of wet cell paste to obtain a 2.5% v/v cell loading (Vortex Genie 2, Scientific Industries, Inc). The cell/latex mixture was pipetted onto each loofah cube and dried at 20 °C for 24 h. After immobilization, each biocomposite was submerged in 2 mL of growth medium and shaken in darkness at 80 RPM for 1, 24, 48 and 72 h. The growth medium was refreshed at each stated time interval and any cells released from the loofah surface were counted using the hemocytometer and converted to a percentage of the cells loaded onto the loofah scaffold. The most suitable latexes ^2^ were determined based on toxicity and adhesion data, with a weighing of toxicity to adhesion of 3:2 as cell viability was deemed the more important characteristic.

**Photosynthetic response.** An imaging pulse amplitude modulated-fluorometer (Imaging-PAM M-Series MAXI Version; Walz GmbH) was used to determine key photosynthetic responses of cyanobacteria when exposed to the different latexes ^3^. For each sample, 1 mL of the polymer emulsion was combined with 1 mL of concentrated cell mixture to form a biocoating (2.5% v/v cells/ sterile dH_2_O) in individual wells (n = 3) of a 24-well multiplate. The corresponding abiotic controls (latex only) were run in the same well plate. The samples were monitored daily for seven days, during which they were gently mixed using forward and reverse pipetting and left to dark adapt for 30 mins before each record. To mitigate for the high cellular phycocyanin content, the IPAM settings were adjusted to the gain values of 20 to 25 depending on the base intensity and a low saturation pulse intensity of 1. These settings were determined experimentally prior to the assays. The maximum photosystem II (PSII) quantum yield (F_v_/F_m_) was determined from the dark fluorescence yield (F_o_) and maximum fluorescence yield (F_m_) using Equation 1 (with worked example) ^3^. The apparent rate of photosynthesis (PS) was calculated using Equation 2 from the measured effective PSII quantum yield (Y(II)), the incident photon flux density (PAR) which was fixed at 370 µmol photon m^-2^ s^-1^, and the measured absorptivity (Abs.) ^3^. For the calculation of PS, 50% of the absorbed PAR was assumed to be distributed to PSII ^3^.

$$\frac{F_{v}}{F_{m}}= \frac{F_{m}- F_{o}}{F_{m}} (1)$$

An example of how these values were calculated for any given data point is provided below:

Example raw data for replicate 1, 0N for *S. elongatus* PCC 7942:

F*_m_* = 0.4064

F*_o_* = 0.3549

The mean values for the corresponding blank samples (three replicates with no cells) on the same row of well plate were:

F_m,blank_ = 0.0913

F_o,blank_ = 0.0913

The data are normalized to the blanks by:

$$F_{m,sample}-F_{m,blank}=0.4064-0.0913=0.3151$$

$$F_{o,sample}-F_{o,blank}=0.3549-0.0913=0.2636$$

F*_v_*/F*_m_* is then calculated using equation 1:

$$\frac{F_{v}}{F_{m}}= \frac{F_{m}- F_{o}}{F_{m}}$$

$$\frac{F_{v}}{F_{m}}= \frac{0.3151- 0.2636}{0.3151}=0.1635$$

$$PS=0.5 \times Y\left( II \right)\times PAR \times Abs. (2)$$

**Biocomposite microstructure analysis.** Biocomposites were analyzed before and after the CO_2_ absorption tests using scanning electron microscopy (Tescan Vega 3LMU) with a voltage of 8 kV at ×5000 magnification. Biocomposites were dried at 105 °C for 3 h, stored in a desiccator for at least 24 h, and attached to 12 mm diameter pin stubs using carbon tape. Prior to analysis, each sample was gold coated to protect the structure from the electron bombardment and to increase sample conductivity.

**Total carbohydrate extraction.** The total carbohydrate extraction was modified from Moheimani, et al. ^4^. Prior to extraction, the biocomposites from the semi-batch CO_2_ absorption tests were frozen at -20 °C, freeze-dried (Martin Christ 1-4 LD Plus) for 48 h, and ground (Cookworks Coffee and Herb Grinder). Each sample was homogenized with 1 mL of 1M H_2_SO_4_ in a 45 mL acid resistant centrifuge tube then 10 mL of the same solution was added. The samples were incubated in a water bath at 100 °C for 1 h and left to cool to ambient temperature (20 °C). The samples were isolated from the acid solution by centrifugation (Sigma Laboratory Centrifuges, 3K18 C) at 2000 RCF for 10 mins. For each sample, 2 mL was carefully transferred into a new centrifuge tube without disturbing the solid residue. The isolated extract was mixed with 1 mL of phenol solution (50 g L^-1^) before 5 mL concentrated H_2_SO_4_ (>95%) was added, and both solutions vortexed for 10 to 20 sec (Vortex Genie 2, Scientific Industries, Inc.). The mixture was left to cool to ambient temperature (20 °C) before 1 mL was placed into a 1.5 mL cuvette and analyzed in a UV-Vis spectrometer (Cary 100 Bio UV-Visible Spectrometer) at a wavelength of 485 nm. A standard calibration curve of known glucose concentrations was used to calculate the total carbohydrate content in the biocomposites. The samples were sacrificially analyzed for carbohydrate content at weeks 2, 4, 6, and 12 of the CO_2_ absorption tests (n = 3 for each treatment). The carbohydrate extraction was also conducted on abiotic controls (i.e., loofah scaffold with latex only) to normalize the biocomposites, and presented as a percentage of the total carbohydrate content in immobilized dry weight biomass.


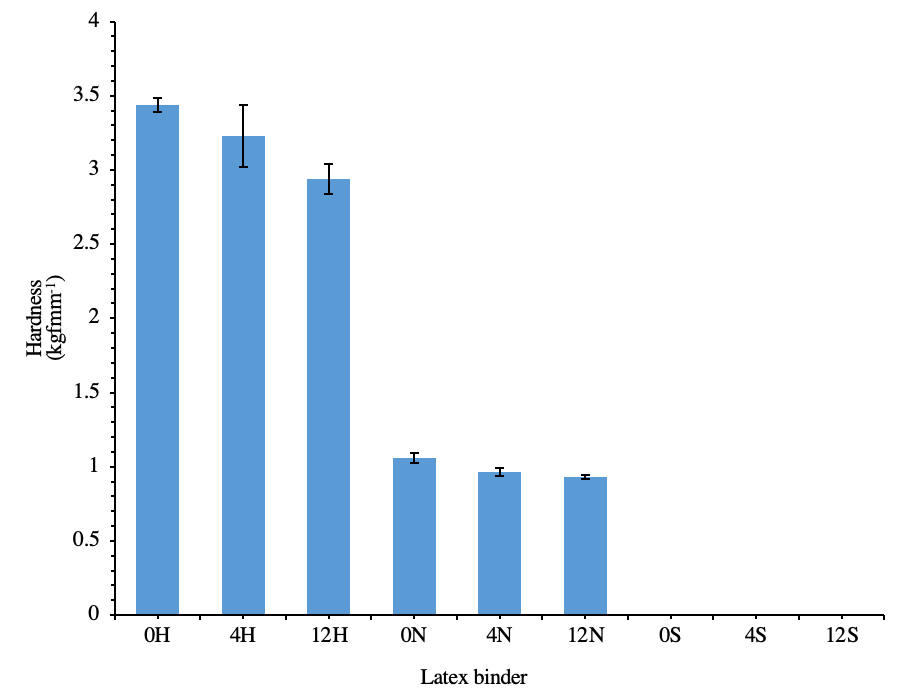


**Figure S1.** The hardness of latex binders as determined by a Buehler Micromet II microhardness tester. H = hard latex, styrene:butyl acrylate = 1:3; N = normal latex, styrene:butyl acrylate = 1:1; S = soft latex styrene:butyl acrylate = 3:1. Hardness is expressed as the Vickers number. The soft latex binders (0S, 4S, and 12S) could not be measured as the latex exhibited excessive sticking and stretching during testing. The numeric value in the binder code refers to the Texanol content (% v/v).


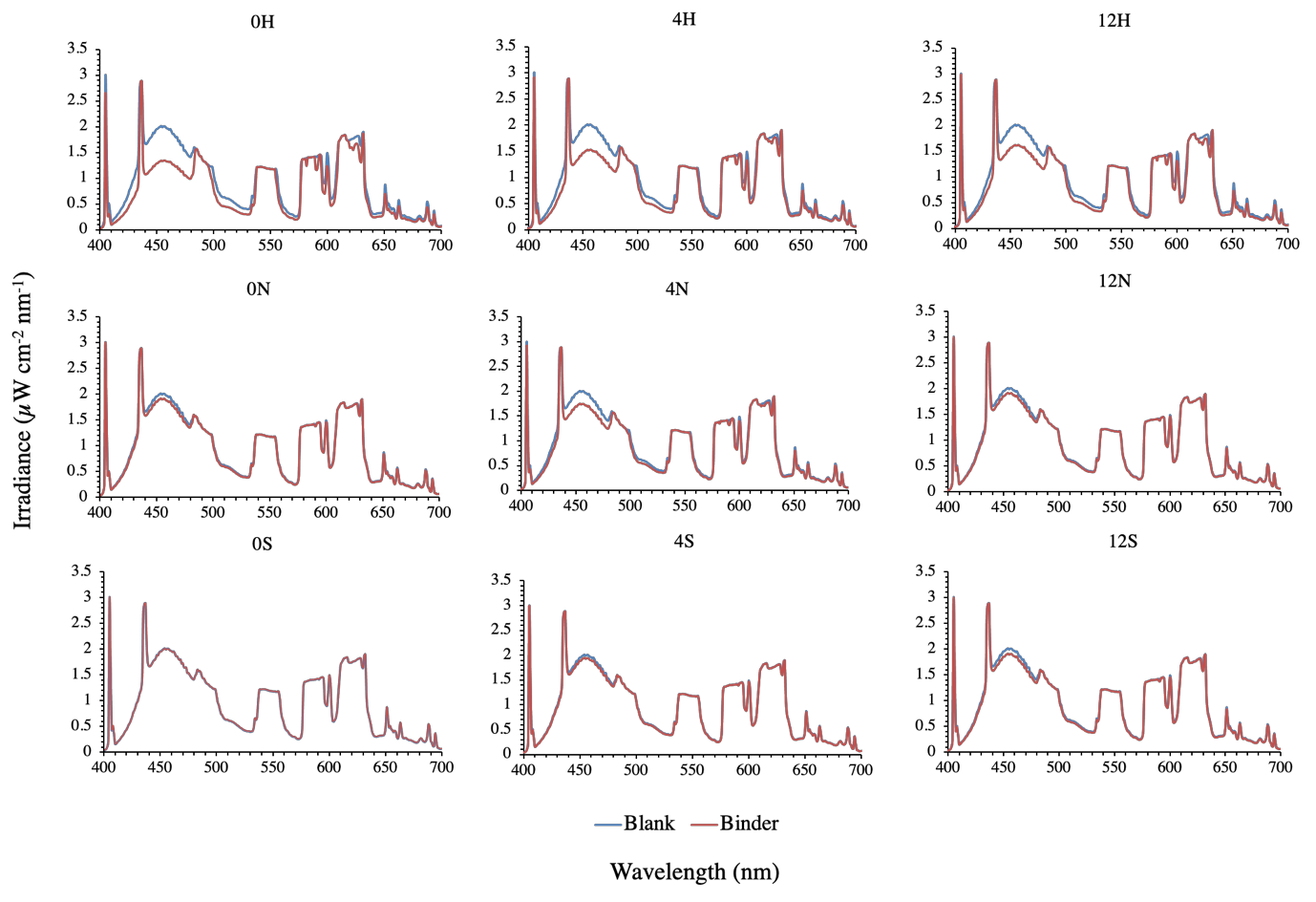


**Figure S2.** Transmission of photosynthetically active radiation wavelengths (400-700 nm) through latex binders coated on glass microscope slides (red lines) versus microscope slide blanks (blue lines). H = hard latex, N = normal latex, S = soft latex. The numeric value in the binder code refers to the Texanol content (% v/v).


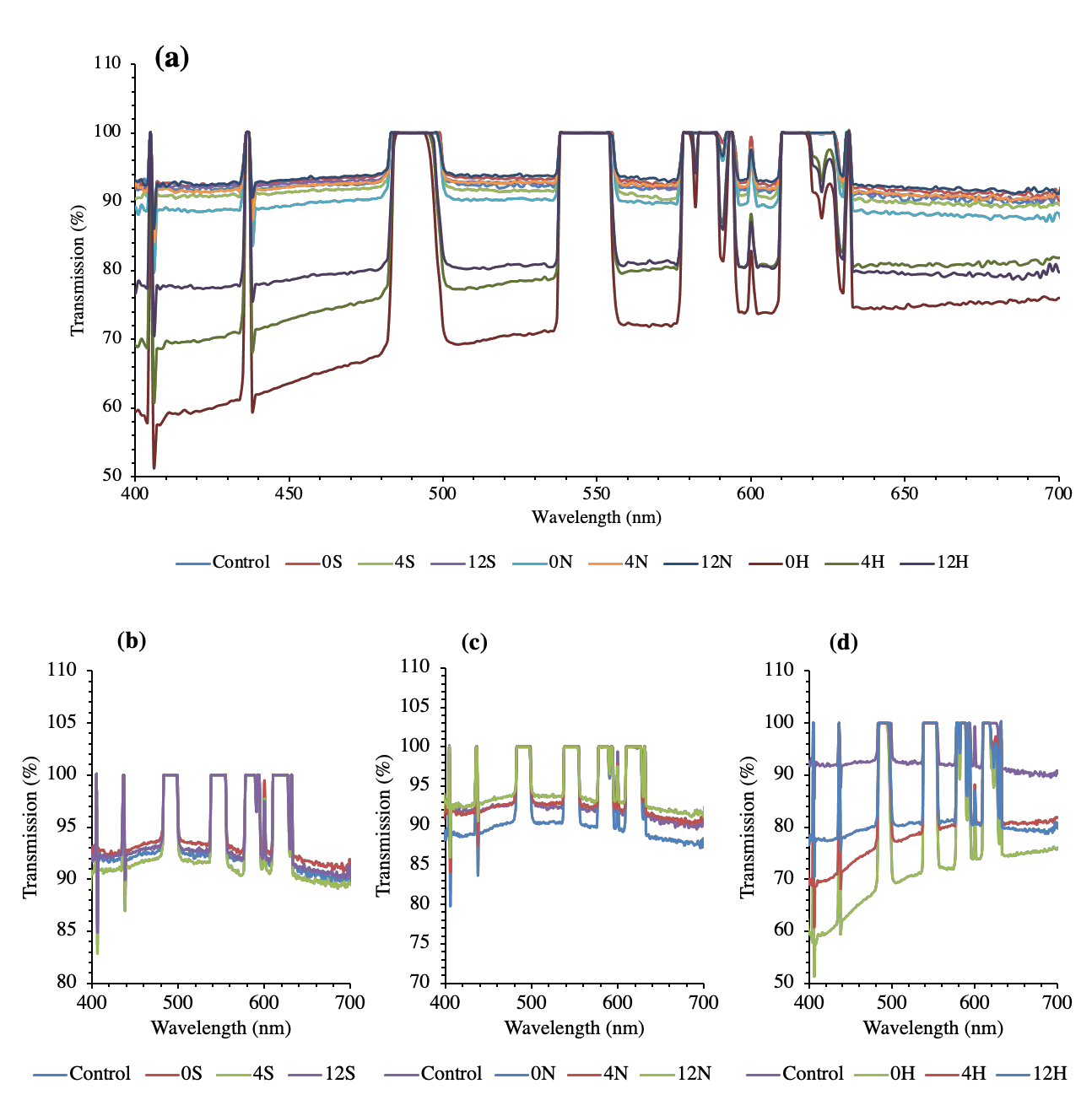


**Figure S3.** Transmission of photosynthetically active radiation wavelengths (400-700 nm) through latex binders coated on glass microscope slides (red lines) versus microscope slide blanks (blue lines). H = hard latex, N = normal latex, S = soft latex. The numeric value in the binder code refers to the Texanol content (% v/v).


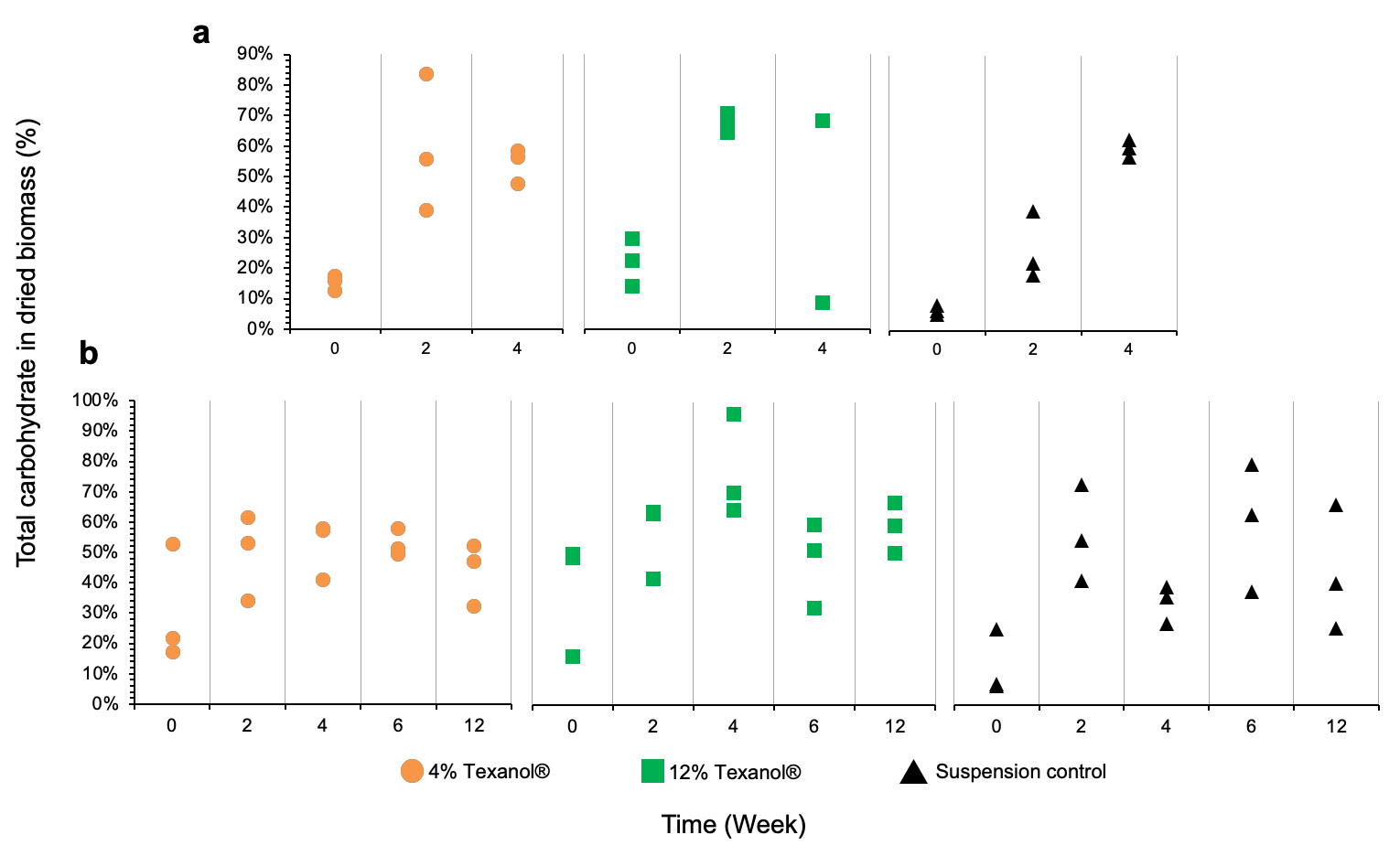


**Figure S4.** **Total carbohydrate content extracted from *Synechococcus elongatus* biocomposites** **during the semi-batch CO_2_ absorption tests compared with suspension culture controls.** **a** *S. elongatus* PCC 7942 with ‘normal’ latex biocomposites. **b** CCAP 1479/1A with ‘soft’ latex biocomposites. ‘Normal’ latex had a styrene to butyl acrylate ratio of 1:1, and ‘soft’ was 3:1.

1 In-na, P. *et al.* Loofah-based microalgae and cyanobacteria biocomposites for intensifying carbon dioxide capture. *J. CO2 Util.* **42**, 101348, (2020).

2 Kosky, P., Balmer, R., Keat, W. & Wise, G. in *Exploring Engineering* (eds Philip Kosky, Robert Balmer, William Keat, & George Wise) 383-396 (Academic Press, 2013).

3 GmbH, H. W. *Imaging-PAM M-series chlorophyll fluorometer: Instruction description and information for users*. (2019).

4 Moheimani, N. R., Borowitzka, M. A., Isdepsky, A. & Sing, S. F. in *Algae for Biofuels and Energy* 265-284 (2013).
